# Associations between livestock ownership and lower odds of anaemia among children 6–59 months old are not mediated by animal‐source food consumption in Ghana

**DOI:** 10.1111/mcn.13163

**Published:** 2021-03-01

**Authors:** Nathalie J. Lambrecht, Mark L. Wilson, Ana Baylin, Gloria Folson, Samuel Naabah, Joseph N. S. Eisenberg, Bright Adu, Andrew D. Jones

**Affiliations:** ^1^ Department of Nutritional Sciences, School of Public Health University of Michigan Ann Arbor Michigan USA; ^2^ Department of Epidemiology, School of Public Health University of Michigan Ann Arbor Michigan USA; ^3^ Department of Nutrition, Noguchi Memorial Institute for Medical Research University of Ghana Accra Ghana; ^4^ Department of Immunology, Noguchi Memorial Institute for Medical Research University of Ghana Accra Ghana

**Keywords:** anaemia, animal‐source foods, child morbidity, Ghana, inflammation, iron deficiency, livestock

## Abstract

Livestock ownership may mitigate anaemia among young children by providing access to animal‐source foods (ASFs) yet exacerbate anaemia by exposing children to animal‐source pathogens. This study aimed to assess the association between household livestock ownership and child anaemia and examine whether this relationship is mediated by child ASF consumption or by child morbidity and inflammation. We conducted a cross‐sectional study of 470 children aged 6–59 months in Greater Accra, Ghana. Child blood samples were analysed for haemoglobin concentration, iron status biomarkers and inflammatory biomarkers. Caregivers were asked about the child's frequency of ASF consumption in the past 3 months. Livestock ownership was categorized into five typologies to distinguish households by the number and combinations of species owned. In adjusted logistic regression, children from households in Type 5, owning cattle, small livestock (goats, sheep or pigs) and poultry, had lower odds of anaemia compared with those in Type 1, owning no livestock (OR [95% CI]: 0.32 [0.14, 0.71]). Although children from households that owned poultry were more likely to consume chicken meat, and children from households with cattle were more likely to drink cow's milk, consumption of these ASFs did not mediate the observed association between livestock ownership and child anaemia. There were no associations between livestock ownership and children's symptoms of illness or inflammation. Further research is needed to understand how ownership of certain livestock species, or a greater diversity of livestock species, may be associated with the risk of child anaemia, including the role of dietary and income‐based pathways.

**Key messages**

- Children 6–59 months old from households that owned cattle had lower odds of anaemia, but not of iron deficiency, compared with children from non‐livestock‐owning households.
- Among livestock owners, having more total livestock and more goats, sheep or pigs was associated with lower odds of anaemia in children.
- Chicken meat and cow's milk were more commonly consumed among poultry‐ and cattle‐owning households, respectively, but consumption of these foods did not mediate the association between livestock ownership and lower odds of anaemia.
- Household livestock ownership was not associated with diarrhoea, fever, illness or elevated inflammation in young children.

## 1 INTRODUCTION

Anaemia affects over half of all children aged 6–59 months in sub‐Saharan Africa and South East Asia (World Health Organization, 2017a). Anaemia in children is associated with severe consequences, including deficits in motor, cognitive, and social‐emotional development (Lozoff, 2007; McCann & Ames, 2007) and increased risk of child mortality (Scott et al., 2014). The causes of anaemia are complex and interconnected, including several micronutrient deficiencies, infectious diseases and genetic haemoglobin (Hb) disorders (Balarajan et al., 2011; Chaparro & Suchdev, 2019). Whereas addressing the proximal causes of anaemia (e.g. iron deficiency, malaria and hookworm infection) is important for treating and preventing anaemia in endemic regions, equally important is identifying factors that influence the underlying determinants of anaemia and its etiological factors, for instance, food insecurity and unhygienic environments.

**FIGURE 1:**
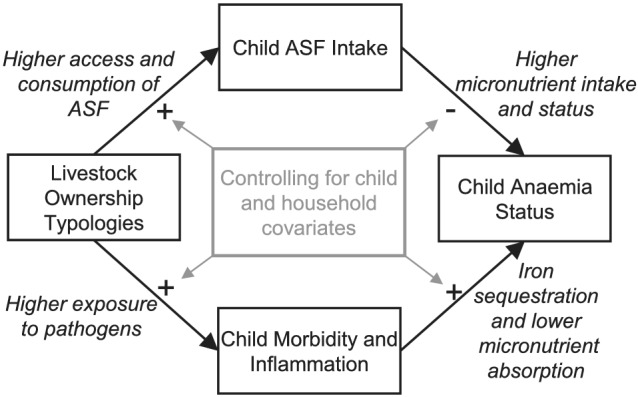
Hypothesized pathways by which livestock ownership influences child anaemia, mediated by ASF intake, morbidity symptoms or inflammation. Livestock ownership typologies were defined as five categories including no livestock; only poultry (<12); only poultry (≥12); small livestock +/− poultry; and cattle +/− small livestock or poultry. Child ASF intake was characterized as consumption of each type of ASF (cow meat; goat, sheep or pig meat; chicken meat; organ meats; eggs; cow milk) in the past 3 months. Child morbidity was considered separately for each type of illness (fever; diarrhoea; cough/cold) experienced in the past 7 days and elevated concentrations of each inflammatory biomarker (CRP > 5 mg/L; AGP > 1 g/L). Anaemia was defined as haemoglobin <11.0 g/dL

Many poor households in low‐ and middle‐income countries (LMICs) keep small numbers of livestock as a source of farm labour, income, insurance and food (Herrero et al., 2013; Randolph et al., 2007). Livestock keeping can improve diets by directly providing animal‐source foods (ASFs), including meat, eggs and milk. ASFs are especially beneficial to young children as they are rich in highly bioavailable micronutrients, particularly in nutrients that affect anaemia—heme iron (high in beef, other red meats, organ meats), vitamin A (meat, eggs) and vitamin B12 (meat, eggs, milk) (Iannotti et al., 2014; Neumann et al., 2002). Utilization of livestock for food in low‐income settings, however, may be minimal among households that keep livestock as a financial asset. Nevertheless, the income from the sale of livestock and livestock products can improve diets if households use this income to purchase ASFs or other nutrient‐rich foods such as leafy greens and vitamin A‐rich fruits and vegetables. Conversely, livestock faeces are a likely source of infection and illness for children in LMICs (Penakalapati et al., 2017; Prendergast et al., 2019). Faecal contamination from livestock is high where sanitation resources are poor and livestock, particularly poultry, free‐roam and cohabitate the household environment (Ercumen et al., 2017; Fuhrmeister et al., 2019). Young children, especially those under 2 years old who engage in hand‐to‐mouth behaviour, may be exposed to livestock faeces through various pathways, including touching uncontained faeces (Ngure et al., 2013) and ingesting contaminated soil, water and food (Penakalapati et al., 2017). The pathogens that livestock carry, including soil‐transmitted helminths and enteropathogenic bacteria (Anchang et al., 2014; Delahoy et al., 2018), can lead to anaemia through blood loss and inflammation‐induced iron sequestration in cells (Jonker et al., 2017). Repeated exposure to enteropathogens may also reduce iron absorption via environmental enteric dysfunction (EED), an inflammatory condition that is common in young children living in LMICs, and is associated with intestinal permeability, intestinal and systemic inflammation and nutrient malabsorption (Watanabe & Petri, 2016). Because livestock ownership may simultaneously influence children's diets and exposure to infectious pathogens, understanding whether livestock ownership affects anaemia and the pathways through which it may do so is critical in developing programmes that address child anaemia (Figure 1).

Several studies have examined the impacts of livestock ownership on child growth outcomes within this nutrition‐benefit and infection‐risk framework (Azzarri et al., 2015; Choudhury & Headey, 2018; de Bruyn et al., 2018; Dumas et al., 2018; Fierstein et al., 2017; Headey et al., 2017; Headey & Hirvonen, 2016; Hetherington et al., 2017; Hoddinott et al., 2015; Jin & Iannotti, 2014; Kaur et al., 2017; Mosites et al., 2015, 2016), yet few have robustly examined impacts on anaemia in young children, and thus far, findings have been mixed (Lambrecht et al., 2019). Intervention studies promoting poultry production have shown modest reductions or no changes in anaemia among children under 5 years of age (Hillenbrand & Waid, 2014; Olney et al., 2009, 2015; Osei et al., 2015, 2017; Passarelli et al., 2020; Talukder et al., 2014). Most of these interventions, however, combined poultry production with home gardening and behaviour change, thus making it difficult to identify benefits derived specifically from livestock rearing. Further, agricultural nutrition‐sensitive interventions that aim to influence several domains across programme impact pathways (i.e. household food production, diets and nutritional status) may be underpowered to detect changes in nutritional status within the study time frame, though sufficiently powered to detect impacts on dietary targets (Herforth & Ballard, 2016). Conversely, three observational studies found that chicken ownership was associated with higher odds of anaemia (two in Ghanaian preschool‐aged children [Christian et al., 2019; Jones et al., 2018] and one in Haitian school‐aged children [Iannotti et al., 2015]). Both studies conducted in Ghana postulated that exposure to pathogenic bacteria in chicken faeces may contribute to the higher likelihood of anaemia in children; however, these pathways were not analysed.

There are still many unknowns regarding the relationships between livestock ownership and child anaemia, including the relative importance of the species and quantity of livestock owned. Furthermore, many households simultaneously own several different animal species, particularly in low‐resource settings, where households incrementally diversify from chickens to larger livestock. These different ownership patterns may influence how households use livestock for income or food, because poultry may be more readily sold and consumed than large‐value livestock assets (Nyantakyi‐Frimpong et al., 2018). For example, a household with a few chickens may be less likely to slaughter its chickens than a household with the stability of higher value livestock assets, such as goats in addition to chickens. Additionally, studies have found species‐specific associations of owning more livestock with contamination of drinking water (Wardrop et al., 2018) and child diarrhoea (Ercumen et al., 2020), though it is unclear if owning multiple species affects exposure or susceptibility to infections.

Accordingly, we assessed the relationship between household livestock ownership and anaemia in young children in Greater Accra, Ghana, and determined whether this relationship was mediated by child ASF consumption or morbidity and inflammation. We categorized typical livestock ownership patterns using a livestock ‘typologies’ approach (de Bruyn et al., 2018; Dumas et al., 2018) in order to examine the effects of owning multiple livestock species. We also examined iron status and inflammation biomarkers, and child diet and morbidity symptoms, to explore different pathways in a low‐income, high‐infection context. Our hypothesis was that livestock ownership, particularly of chickens, would be positively associated with child anaemia, mediated by morbidity and elevated inflammation, and that household livestock and livestock by‐products would be kept as an income‐generating asset and therefore would not substantially contribute ASFs to children's diets.

## 2 METHODS

### 2.1 Study area

This cross‐sectional study was conducted in the Ga East and Shai Osudoku Districts in the Greater Accra Region, Ghana, during October and November 2018 (Figure S1). The Greater Accra Region is located along Ghana's southern border and home to Ghana's capital city of Accra. The Ga East District is primarily urban with most of its working population engaged in service and sales work (Ghana Statistical Service, 2014a), whereas Shai Osudoku District, the largest and least populated district in the Greater Accra Region, is predominantly agrarian (Ghana Statistical Service, 2014b). In 2014, anaemia prevalence among 6‐ to 59‐month‐old children in the Greater Accra Region was 59.6% (Ghana Statistical Service, Ghana Health Service, and ICF International, 2015).

### 2.2 Study design

The study population was children 6–59 months of age. Inclusion criteria for households to participate in the study were (1) the household had at least one child aged 6–59 months old and (2) the primary caregiver for that child had to be 18 years or older.

The study sample size was based on detecting a difference of 0.4 g/dL Hb concentration between children living in livestock‐ and non‐livestock‐owning households using an independent two‐sample *t*‐test with a statistical significance level of 0.05 and a power of 0.80, giving us a minimum sample size of 466 children. However, post hoc analyses using a typologies‐based approach were underpowered (<50% power) to detect a difference in child anaemia.

Eighteen livestock‐rearing communities in the Ga East (*n* = 8) and Shai Osudoku (*n* = 10) districts were purposefully selected to maximize variation in community size and types of livestock reared. The study team conducted a household census in each community to identify which households had at least one child 6–59 months of age and to gather initial information on household livestock ownership. There were fewer 6‐ to 23‐month‐old children in the census compared with 24‐ to 59‐month‐old children. Thus, to obtain a sample with an equal number of children in these age groups, enumerators recruited from all possible households with children 6–23 months old first, and then a random sample of households with children 24–59 months old, up to 40 households total per community. In households with more than one child between 6 and 59 months old, the youngest child was selected for recruitment.

### 2.3 Data and specimen collection

Six enumerators were trained in the study protocols through classroom and field sessions during 1 week before the start of the study. Enumerators agreed upon translations of the survey from English to local languages (Twi, Ga, Ewe, Dangme). Survey data were collected using electronic tablets (Samsung Galaxy Tab A, Model Number SM‐T285) with the Qualtrics survey platform (Qualtrics, Provo, UT, USA). The study survey was piloted in two non‐study communities in July 2018.

Enumerators interviewed the primary caregiver, usually the mother, about household socio‐demographic characteristics, livestock ownership and management (Zezza et al., 2016) and the child's diet and health. To assess the child's diet, caregivers were asked about the child's consumption of liquids and foods in the preceding day or night (World Health Organization, 2010a) and were also given a qualitative 3‐month food frequency questionnaire assessing consumption of various ASFs. The food frequency questionnaire asked whether the child had consumed, in the past 3 months, various flesh foods (e.g. beef, goat, chicken and organ meats), milk and milk products, eggs and other ASFs (e.g. snails), as well as how often these foods were consumed (with categories ranging from less than one time per month to two or more times per day). The primary way that each consumed ASF was obtained in the past 3 months (e.g. own production, hunting or fishing or purchased) was also recorded. To evaluate the child's health, caregivers were asked to recall illness symptoms during the previous 7 days, including fever, cough/cold or diarrhoea (≥3 loose stools in 24 h), and treatments such as iron or vitamin A supplements, or intestinal worm treatment.

Blood samples from children were collected via a standard finger prick (World Health Organization, 2010b). Hb concentration was measured in the field using a HemoCue Hb 201 + portable haemoglobinometer (HemoCue AB, Sweden). HemoCue instruments were checked weekly against low, normal and high Haemoglobin Extended Controls (R&D Systems, Minneapolis, MN). Malaria parasitaemia was measured by an antigen rapid diagnostic test (RDT), SD Malaria Ag P.f (HRP2/pLDH) (Standard Diagnostics Inc., Korea). Approximately 200 uL of blood was collected in a Microvette clotting activator/serum tube (Sarstedt, Germany). Samples were transported at room temperature to Noguchi Memorial Institute for Medical Research (Accra, Ghana) and immediately spun in a microcentrifuge at 5000 rpm for 10 min. Then, 50 uL of serum was aliquoted into a 0.2‐mL PCR tube (Sarstedt, Germany) and stored at −20°C. Serum samples were shipped on dry ice to the VitMin Laboratory (Willstaett, Germany) for analysis of serum ferritin (SF), serum transferrin receptor (sTfR), retinol‐binding protein (RBP), C‐reactive protein (CRP) and α‐1‐acid glycoprotein (AGP) using a sensitive sandwich enzyme‐linked immunosorbent assay (ELISA) technique (Erhardt et al., 2004).

Caregivers were instructed to collect the child's morning stool in a sterile faecal container (Sarstedt, Germany). Stool samples were transported on wet ice to the Noguchi Memorial Institute for Medical Research every afternoon. Upon arrival at the laboratory, two trained technicians independently analysed stool samples using the Kato–Katz method for eggs of the helminths *Ascaris lumbricoides*, *Trichuris trichiura*, *Ancylostoma duodenale* and *Necator americanus* (World Health Organization, 1994).

### 2.4 Variable definitions

The primary independent variable in this study was household livestock ownership, defined as owning or rearing cattle, goats, sheep, pigs, chickens, ducks, turkeys, guinea fowl, grasscutter, rabbits or rats. We developed a livestock typologies indicator based on the methods used in Dumas et al. (2018) and de Bruyn et al. (2018) to categorize typical livestock ownership patterns within the study sample. Most of the households in our sample owned chickens, so we created two categories for poultry ownership to distinguish households that owned more and less than the median number of poultry. Five typology categories were created:

- Type 1—No livestock;
- Type 2—Poultry only, less than 12;
- Type 3—Poultry only, 12 or more;
- Type 4—Small livestock (goats, sheep, pigs), rodents (grasscutter and other rats) or rabbits, with or without poultry;
- Type 5—Cattle, with or without small livestock or poultry.

We examined additional indicators of livestock ownership to parse out effects due to ownership of specific livestock species and the quantity of livestock owned and for comparability with other studies examining effects of livestock ownership on child nutrition outcomes. These indicators included dichotomous ownership of any livestock and distinct livestock species; number of animals owned in total and by species; and tropical livestock unit (TLU) score. TLU is a commonly used indicator to standardize each household's total livestock assets, accounting for the weight and market value of different livestock species (Njuki et al., 2011). TLU was calculated as the sum of the number of each livestock species multiplied by its weight conversion factor (0.70 for cattle; 0.20 for pigs; 0.10 for goats and sheep; 0.03 for ducks, turkeys and guinea fowl; 0.02 for grasscutter, rabbits and rats; and 0.01 for chickens). Continuous variables were log‐base two transformed to normalize the distribution of animals owned by livestock‐owning households.

The primary outcome variable was child anaemia, defined as Hb < 11.0 g/dL for children 6–59 months old (World Health Organization, 2011). Iron deficiency, analysed as a secondary outcome, was defined as low SF (SF < 12 μg/L), indicative of low iron stores, or high sTfR (sTfR >8.3 mg/L), indicative of low availability of iron to tissues (Namaste et al., 2017). SF concentrations were adjusted for inflammation (CRP and AGP) using the regression correction approach outlined by Namaste et al. (2017).

Child diet and morbidity/inflammation were analysed as mediators to examine the two hypothesized pathways from livestock ownership to child anaemia. The child's diet was assessed using three metrics: (1) a dietary diversity score ranging from 0 to 7, calculated as the sum of seven food groups consumed in the previous 24 h (World Health Organization, 2010a); (2) intake of ASF categories in the past 24 h (cow meat, other red meat, chicken or other poultry meat, organ meats, eggs, animal milk); and (3) intake of ASF categories in the past 3 months (cow meat, goat, sheep, or pig meat, chicken or other poultry meat, organ meat, eggs, fresh cow milk). The number of times per month that ASF categories were consumed during the prior 3 months was also calculated, ranging from 0 to 62 times per month (if the food group was consumed at least twice per day in a 31‐day month). Child morbidity indicators included whether or not the child had fever, diarrhoea or a cough or cold in the 7 days before the survey and whether the child had inflammation, defined as CRP > 5 mg/L or AGP > 1 g/L (Namaste et al., 2017).

Several socio‐demographic and child‐level covariates, identified *a priori* as theoretical determinants of anaemia (Engle‐Stone et al., 2017; Shenton et al., 2020; SPRING, Ghana Health Service, 2016), were collected. Household‐level socio‐demographic variables included the number of children under 5 years old; the mother's education level; the household head's sex, religion and ethnic group; and the household's access to improved water (World Health Organization, 2017b), sanitation and an asset‐based wealth index. The household wealth index was created using principal component analysis (PCA) of 26 assets (e.g. electricity and radio), excluding land holdings and livestock assets (Vyas & Kumaranayake, 2006), with quintiles of the first component being used as the asset score. Child‐level covariates included child sex, age, breastfeeding status and malaria parasitaemia. The child's age, based on date of birth, was verified through clinical child health records. For three children whose health records were unavailable, but for whom we had data on their month and year, but not day of birth, we imputed that the child was born on the 15th of that month.

### 2.5 Statistical analysis

Data cleaning and statistical analysis were conducted using Stata SE Version 14.2 (StataCorp, College Station, TX, USA). Mediation analysis was done using the mediation package in R (R Core Team, 2019; Tingley et al., 2014).

Descriptive analyses of child‐ and household‐level characteristics in the overall sample, and by child anaemia status and household livestock typologies, were conducted. We used logistic regression to model the association between livestock ownership typology and anaemia. The adjusted models controlled for the child‐ and household‐level covariates described above and for study district fixed effects. Child breastfeeding status was excluded from the adjusted models as it was strongly associated with child age. Improved water source was also excluded as there was little variation and its inclusion did not alter effect estimates.

As a sensitivity analysis, adjusted associations between additional indicators of livestock ownership and anaemia were analysed using logistic regression. We also analysed the associations between livestock ownership indicators and Hb concentration using linear regression and iron deficiency using logistic regression. To test whether younger children may be at higher anaemia risk from livestock exposure, we ran an adjusted logistic regression model with an interaction term between livestock typology and child age (6–23 months vs. 24–59 months). In all of the regression models, we calculated robust standard errors clustered at the community level.

Mediation by child diet and morbidity variables was assessed using a two‐step approach. First, we analysed the bivariate associations between livestock ownership typology and the mediators using chi‐square and ANOVA tests. For relationships that showed evidence of an association (*P* < 0.05) between livestock ownership and one of the mediators, the R mediation package was used to assess the total, direct and indirect effects of typology on anaemia through that mediator (Tingley et al., 2014). Two logistic models were specified: the mediation model, which predicted the mediator conditional on livestock typology and select covariates, and the outcome model, which predicted anaemia conditional on livestock typology, the mediator and covariates. The average causal mediated effect (indirect effect) was then estimated for each type in comparison to Type 1 (no livestock owned) using quasi‐Bayesian Monte Carlo with 500 simulations and clustering set at the community level. To further examine associations between livestock ownership typologies and ASF consumption, we used multivariate logistic regression to model the adjusted associations between livestock ownership typologies and ASF consumption in the past 3 months.

Statistically significant associations are reported at the *P* < 0.05 level.

### 2.6 Ethical considerations

This study was approved by the University of Michigan Health Sciences and Behavioural Sciences Institutional Review Board (protocol no. HUM00145171) and the Noguchi Memorial Institute for Medical Research Institutional Review Board (protocol no. 098/17‐18). Before any data were collected, we met with community leaders to explain the project and obtain their general approval. At each household, the child's legal guardian provided written, informed parental consent for their child's participation in the study and the primary caregiver (parent or other adult) provided written informed consent for their participation in the interview. If the parent or caregiver had a low level of literacy, the enumerator read the consent documents aloud in the presence of a witness. If the parent or caregiver consented to participate, they provided a thumbprint and the witness signed the form certifying that the study was explained and the parent or caregiver had agreed to participate. Households were given a non‐monetary gift (e.g. washbasin) as compensation for participating in the study.

## 3 RESULTS

### 3.1 Sample characteristics

In total, 484 households provided informed consent and were included in the study. Of those, we excluded 10 households (2.9%) that had missing information on the mother's education level (*n* = 3), the child's date of birth (*n* = 2), consumption of ASFs (*n* = 2), morbidity symptoms (*n* = 1) or SF and sTfR measurements (*n* = 2). We also excluded four households that were poultry farms (>500 poultry) as children from these households had Hb concentrations 0.5 g/dL higher than the sample average, a greater likelihood of consuming chicken meat and eggs, and were in the highest wealth quintile. The final sample size was 470 households.

Nearly half (47.9%) of children were anaemic (Table 1). Iron deficiency was common, with 52.8% of all children presenting with elevated sTfR and 25.1% presenting with low SF. The prevalence of anaemia was substantially higher among children 6–23 months old than among 24‐ to 59‐month‐olds. Anaemia was associated with iron deficiency and vitamin A deficiency, lower dietary diversity score and malaria parasitaemia.

**TABLE 1:** Child and household characteristics overall and by anaemia status among 6‐ to 59‐month‐old children in Greater Accra region, Ghana, October–November 2018

Indicator	Overall (*n* = 470)	Anaemic (*n* = 225)	Not anaemic (*n* = 245)
Child characteristics			
Female sex, %	52.1	54.2	50.2
Age (6–23 months old), %	50.9	60.9	41.6***
*Health indicators*			
Iron deficiency (SF < 12 μg/L), %	25.1	40.0	11.4***
Iron deficiency (sTfR>8.3 mg/L), %	52.8	71.1	35.9***
Vitamin A deficiency (RBP < 0.70 μmol/L), %	16.0	19.6	12.7*
Inflammation (CRP > 5 mg/L), %	16.0	17.8	14.3
Inflammation (AGP > 1 g/L), %	35.5	38.7	32.7
Fever in past 7 days, %	23.6	24.4	22.9
Diarrhoea in past 7 days, %	7.7	8.9	6.5
Cough/cold in past 7 days, %	22.6	21.8	23.3
Malaria parasitaemia, %	8.5	12.4	4.9**
Helminth infection, % (*n* = 415)	0	0	0
*Dietary indicators*			
ASF consumption in past 24 hours^a^, %	84.9	82.2	87.4
ASF consumption in past 3 months^a^, %	97.7	96.9	98.4
Dietary diversity score	3.4 ± 1.6	3.3 ± 1.6	3.6 ± 1.5*
Household characteristics			
Number of children < 5 years	1.4 ± 0.6	1.4 ± 0.6	1.3 ± 0.5*
Head of household sex (female), %	21.9	24.0	20.0
Head of household religion, %			
Christian	85.7	83.1	88.2
Muslim	11.5	15.1	8.2
Other	2.8	1.8	3.7
Head of household ethnic group, %			
Ga–Dangme	42.8	45.8	40.0
Akan	15.7	16.0	15.5
Ewe	27.7	20.9	33.9
Other	13.8	17.3	10.6
Maternal education, %			
None or nursery	21.3	21.3	21.2
Primary	23.0	25.3	20.8
Junior	41.3	39.1	43.3
Senior or higher	14.5	14.2	14.7
Access to electricity, %	87.5	88.0	86.9
Access to improved water source, %	97.0	97.3	96.7
Type of sanitation, %			
Flush or pour flush	12.6	11.1	13.9
Pit latrine	58.1	60.4	55.9
Open defecation	29.4	28.4	30.2
Child slept under mosquito bed net in previous night, %	59.4	60.9	58.0
Asset‐based wealth quintile, %			
Lowest	19.2	20.9	17.6
Low	20.6	20.4	20.8
Middle	20.9	19.6	22.0
High	20.4	22.2	18.8
Highest	18.9	16.9	20.8
District, %			
Shai Osudoku	58.5	64.9	52.7**
Ga East	41.5	35.1	47.4**
*Livestock*			
Ownership of any livestock, %	43.6	40.9	46.1
Cattle, %	3.2	2.2	4.1
Goats, sheep or pigs, %	14.3	10.2	18.0*
Poultry (chickens, turkeys, ducks or guinea fowl), %	41.1	38.7	43.3
Livestock typology, %			
Type 1—No livestock	56.4	59.1	53.9
Type 2—Poultry (<12)	15.1	16.4	13.9
Type 3—Poultry (≥12)	12.6	12.9	12.2
Type 4—Small livestock +/− poultry	12.8	9.3	15.9
Type 5—Cattle +/− small livestock or poultry	3.2	2.2	4.1
Tropical livestock units	1.7 ± 8.8	1.0 ± 6.3	2.3 ± 10.6
Total number of livestock	10.9 ± 24.3	8.2 ± 18.4	13.3 ± 28.5*

*Notes*: Values are % or mean ± SD. *P*‐values indicate statistical significance for the difference between anaemic and non‐anaemic children using chi‐square tests for categorical variables and *t*‐tests for continuous variables.

Abbreviations: AGP, α‐1‐acid glycoprotein; ASF, animal‐source food; CRP, C‐reactive protein; RBP, retinol‐binding protein; SF, serum ferritin; sTfR, serum transferrin receptor.

^a^ Animal‐source foods (ASF) include meat (beef, other red meat, organ meat, chicken meat, other poultry meat), fish (fresh or dried fish or shellfish), eggs and dairy (milk, cheese, yogurt or other foods made from milk).

* *P* < 0.05.

** *P* < 0.01.

*** *P* < 0.001.

### 3.2 Livestock ownership characteristics

**FIGURE 2:**
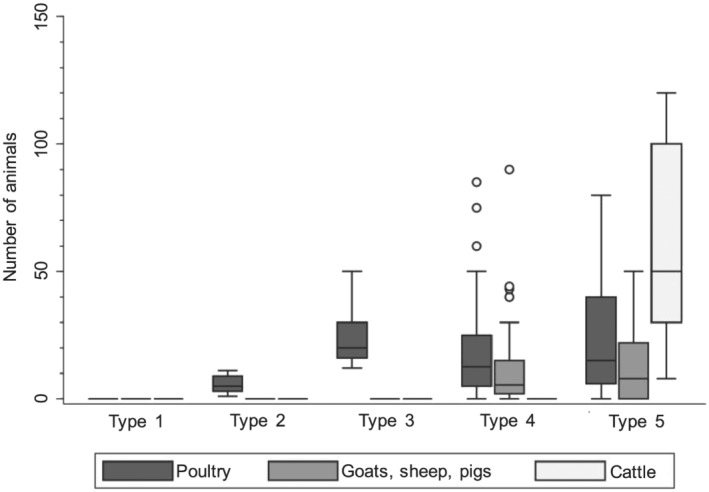
Box plots of the number of animals reared stratified by livestock species and livestock ownership typology among 470 households sampled during October–November 2018 in Greater Accra region, Ghana (265 households were categorized in type 1, 71 in type 2, 59 in type 3, 60 in type 4, and 15 in type 5). Top and bottom whiskers represent the highest and lowest values, top and bottom of boxes represent the 75th and 25th percentiles, middle lines of boxes represent medians, and circles represent outlying values

Overall, 43.6% of households owned or reared livestock, predominantly chickens, either alone or with other types of livestock (Tables 1 and S1). Among Type 2, households owned a median of five chickens, whereas Type 3 households owned a median of 20 chickens (Figure 2 and Table S1). Among households in Type 4, 71.7% owned goats although ownership of sheep and pigs was less common, and most of these households (81.7%) also owned poultry. For the 15 households in Type 5 that reared cattle, 14 also reared chickens, and 10 also reared goats, sheep or pigs. No differences in child characteristics or measures of asset‐based wealth were observed across the livestock typologies, though several household‐level characteristics were different between Type 5 and the other four groups (Table S2). Households in Type 5 had on average more household members, were predominantly Muslim (53.3% compared with 9.1%–13.3% in other typologies) and were of a minority ethnic group. There were no female‐headed households in Type 5 (compared with 11.9%–28.2% in other typologies), and most mothers had no formal education (60.0% in Type 5 households compared with 11.3%–30.5% in households of other typologies). Agricultural labour (i.e. crop farming and/or tending livestock) was the primary occupation of the household head for most Type 5 households (93.3%). This was the primary occupation of fewer than one‐third of households in other typologies (10.9%–33.3%). Access to sanitation facilities also differed across typologies, with 60.0% of Type 5 households practising open defecation compared with 23.4%–35.6% of households in other typologies. Livestock ownership was more common in the more rural Shai Osudoku District than the Ga East District, and all Type 5 households were in Shai Osudoku.

Households kept chickens primarily for feeding the family or for both food and financial purposes (i.e. sale of live animals and income). Goats, sheep and pigs, however, were kept primarily for sale of live animals and income, and occasionally for food, and cattle were kept almost exclusively for income and sale of live animals. Over one‐third of poultry‐owning households reported slaughtering poultry for household consumption, consuming on average three poultry birds in the prior 3 months, whereas less than one‐fifth had sold poultry (Table S3). Approximately 38% of poultry‐owning households reported consuming eggs laid by their poultry, on average seven eggs per week, but rarely sold eggs. Over one‐third of households reported selling larger livestock in the prior 12 months, on average five goats, sheep or pigs among small livestock‐owning households and four cattle among cattle‐owning households. Most cattle‐owning households sold, rather than consumed, the milk produced by their cattle.

### 3.3 Livestock ownership and child anaemia

In unadjusted analyses, children in Type 4 households had lower odds of anaemia compared with children in Type 1 (Table 2). After adjusting for child and household socio‐demographic characteristics, only children in Type 5 households had lower odds of anaemia in comparison with children from households with no livestock (OR [95% CI]: 0.32 [0.14, 0.71]). The association between Type 4 and anaemia was attenuated and marginally significant after adjustment for confounders (0.62 [0.36, 1.07]). We evaluated whether child age modified the association between livestock ownership and anaemia but found no evidence for interaction when comparing children 6–23 and 24–59 months old (*Χ* ^2^ (4) = 5.40, *P* = 0.14).

**TABLE 2:** Unadjusted and adjusted logistic regression of the association of household livestock ownership typology and anaemia in children aged 6–59 months in Greater Accra region, Ghana, October–November 2018 (*n* = 470)

Indicator	Unadjusted model		Adjusted model	
	OR	95% CI	OR	95% CI
Livestock ownership typology (ref: No livestock)				
Type 2—Poultry (<12)	1.08	(0.72, 1.62)	1.14	(0.74, 1.76)
Type 3—Poultry (≥12)	0.96	(0.57, 1.60)	1.07	(0.63, 1.82)
Type 4—Small livestock +/− poultry	0.53*	(0.31, 0.93)	0.62^a^	(0.36, 1.07)
Type 5—Cattle +/− small livestock or poultry	0.50	(0.19, 1.29)	0.32**	(0.14, 0.71)
Child sex (ref: Male)				
Female			1.17	(0.95, 1.44)
Child age (ref: 24–59 months)				
6–23 months			2.56***	(1.68, 3.92)
Child has malaria			3.04**	(1.48, 6.25)
Number of children under 5 years in household			1.52**	(1.17,1.97)
Head of household sex (ref: Male)				
Female			1.13	(0.69, 1.84)
Head of household religion (ref: Christian)				
Muslim			1.73	(0.48, 6.25)
Traditional or no religion			0.79	(0.26, 2.38)
Head of household ethnic group (ref: Ga–Dangme)				
Akan			1.11	(0.65, 1.89)
Ewe			0.51**	(0.33, 0.77)
Other			1.28	(0.52, 3.20)
Maternal education (ref: None or nursery)				
Primary			1.61^a^	(0.96, 2.71)
Junior			1.19	(0.64, 2.23)
Senior or higher			1.30	(0.49, 3.42)
Household sanitation (ref: Open defecation)				
Pit latrine			1.78	(0.61, 5.16)
Flush or pour flush			1.54	(0.83, 2.87)
Asset‐based wealth quintile (ref: Lowest)				
Low			0.85	(0.46, 1.57)
Middle			0.81	(0.50, 1.33)
High			1.05	(0.48, 2.28)
Highest			0.68	(0.31, 1.46)
District (ref: Ga East)				
Shai Osudoku			2.35***	(1.49, 3.70)

*Notes*: Values are OR (odds ratios) and 95% CI (confidence intervals) from multiple logistic regression models between livestock ownership typology and anaemia. Robust standard errors are adjusted for community cluster. Anaemia is defined as haemoglobin <11.0 g/dL.

^a^ Marginal significance (0.05 ≤ *P* < 0.1).

* *P* < 0.05.

** *P* < 0.01.

*** *P* < 0.001.

Other possible associations were examined between livestock ownership (overall, by species and by quantity) and child health (anaemia, Hb concentration and iron deficiency) (Table S4). There were no associations between livestock ownership overall and anaemia, or by species and anaemia. However, among livestock owners, a twofold increase in the number of total livestock owned, in the number of cattle owned and in the number of goats, sheep or pigs owned was associated with 20%, 14% and 14% lower odds of anaemia in children, respectively. In line with these results, for every twofold increase in TLU score, children had 33% lower odds of anaemia. Ownership of goats, sheep or pigs was associated with a 0.39 g/dL higher Hb concentration, whereas owning more livestock in total was marginally associated with higher Hb concentrations. None of the livestock indicators was associated with iron deficiency.

### 3.4 Livestock ownership and child ASF consumption

Overall, 84.9% of children consumed ASFs (including fish) in the past 24 h, and 97.7% of children consumed ASFs at least once in the previous 3 months (Table 1). There was no difference among the livestock ownership typologies in the consumption of meat, eggs or milk in the past 24 h, nor in children's dietary diversity score (S5). In the 3 months preceding the survey, few children consumed meat from sheep, pigs or rodents or eggs from ducks or guinea fowl. Chicken meat and chicken eggs were consumed by most children, on average, about once per week for chicken meat and twice per week for chicken eggs. Children in Type 3 were 3.5 times more likely to have consumed chicken meat in the past 3 months compared with non‐livestock‐owning households (Figure S2 and Table S6). Livestock‐owning households were not more likely to consume chicken eggs. Children from households in Types 3, 4 and 5 were more likely than children in non‐livestock‐owning households to have consumed fresh cow's milk. Among households with cattle, just under half of the children had consumed fresh cow's milk in the past 3 months, on average, about three times per week.

### 3.5 Livestock ownership and child morbidity

Overall, 23.6% of children experienced a fever in the past 7 days, 7.7% had diarrhoea, and 22.6% had a cough or cold (Table 1). Sixteen per cent of children had elevated concentrations of CRP, and 35.5% had elevated concentrations of AGP. No soil‐transmitted helminth infections were detected in children. Neither illness symptoms nor inflammation was significantly different between the livestock ownership typologies in bivariate analyses, though there was a slightly higher proportion of children with fever among Type 2 and a lower proportion with diarrhoea among Types 4 and 5 (Table S5).

### 3.6 Mediation analysis

Chicken meat and cow milk consumption were the only hypothesized mediator variables significantly associated with livestock ownership typology, but no mediating effects were observed in the association between livestock ownership typologies and anaemia (Table 3).

**TABLE 3:** Estimates of the average causal mediated effect (indirect effect) of chicken meat and cow milk consumption in the association between livestock ownership typology and anaemia among children 6–59 months old in Greater Accra, Ghana (*n* = 470)^a^

Indicator	Chicken meat^b^			Cow milk		
	ACME	95% CI	*P*‐value	ACME	95% CI	*P*‐value
Livestock typology						
Type 1—No livestock (reference)						
Type 2—Poultry (<12)	0.0008	(−0.007, 0.01)	0.82	−0.0005	(−0.0052, 0.00)	0.82
Type 3—Poultry (≥12)	0.0107	(−0.005, 0.03)	0.18	−0.0023	(−0.0188, 0.02)	0.76
Type 4—Small livestock +/− poultry	0.0058	(−0.0046, 0.02)	0.30	−0.0017	(−0.0152, 0.01)	0.77
Type 5—Cattle +/− small livestock or poultry	−0.0068	(−0.0244, 0.00)	0.25	−0.0026	(−0.0308, 0.02)	0.77

^a^ R mediation package (Tingley et al., 2014) was used to estimate the average causal mediated effect (ACME) of chicken meat and cow milk consumption on anaemia for each typology in comparison to Type 1 (no livestock) using quasi‐Bayesian Monte Carlo with 500 simulations and clustering set at the community level. Covariates in mediation models: child age (binary), child sex, number of children under 5, head of household sex, head of household religion, head of household ethnic group, maternal education category and asset‐based wealth quintile. Covariates in outcome models: child age (binary), child sex, number of children under 5, head of household sex, head of household religion, head of household ethnic group, maternal education category, asset‐based wealth quintile, malaria and toilet facility.

^b^ The category for chicken meat includes consumption of other poultry meats (turkey, guinea fowl and duck meat) in the past 3 months. 99.7% of children consumed chicken meat, 2.1% of children consumed other poultry meat in addition to chicken meat, and 0.3% of children consumed other poultry meat but not chicken meat in the past 3 months.

## 4 DISCUSSION

Livestock ownership may mitigate anaemia among young children by providing a source of micronutrient‐rich ASFs, yet concurrently exacerbate anaemia by exposing children to animal‐based pathogens that cause illness and inflammation. Contrary to our hypothesis that livestock ownership would be associated with higher odds of anaemia mediated by symptoms of morbidity and inflammation, we found no negative associations between livestock ownership and child anaemia. Rather, in this cross‐sectional study of 6‐ to 59‐month‐old children in Greater Accra, Ghana, children from households that owned cattle concurrently with small livestock (goats, sheep or pigs) or poultry had lower odds of anaemia than children from households without livestock. Among livestock owners, owning more cattle or small livestock was also associated with lower odds of anaemia. Although some livestock ownership typologies were associated with the consumption of specific ASFs, consumption of those ASFs did not mediate the association between livestock ownership and anaemia. Owning livestock did not necessarily translate to more ASF consumption, and less than half of households consumed meat or eggs from their livestock. Furthermore, despite a clear association between iron deficiency and anaemia, there were no associations between livestock ownership and iron deficiency.

In LMICs, livestock are an important financial resource (Herrero et al., 2013), and households diversify their livestock holdings to utilize the advantages of each species. Poultry are affordable, have short reproductive cycles and are readily sold or exchanged to meet regular household needs (Alders et al., 2018; Wong et al., 2017). Goats, sheep and pigs are insurance that can be used to meet more substantial expenses while still being relatively affordable for households to maintain (Otte et al., 2012). In contrast, large livestock, such as cattle, are more expensive to maintain yet are the most financially valuable (Otte et al., 2012). The livestock indicators that were associated with lower odds of anaemia in this study were those that represent higher livestock assets and greater opportunity for supplemental income from those livestock. Households in our study reported keeping livestock, particularly cattle, goats, sheep and pigs, for financial purposes, and over one‐third of households reported selling live animals in the past year. Among livestock owners, owning more total livestock in addition to owning several types of livestock may also enable more flexible utilization of livestock to meet household needs. Qualitative studies conducted in Ghana have similarly found that households keep livestock foremost as a bank account to be used to pay for school fees and healthcare costs (Aboe et al., 2006; Nyantakyi‐Frimpong et al., 2018).

How financial capital derived from owning large and small livestock species, as well as greater quantities of livestock, may translate into reduced anaemia warrants further investigation, and several plausible pathways may be considered. First, owning livestock may enable households to access and utilize medical care for children who are ill. Second, children from households that can afford school fees may be more likely to receive healthcare services and nutritious meals provided at schools. Third, livestock‐derived income may enable households to increase overall expenditures on food, improving children's nutrition. Alternatively, associations between lower anaemia and larger, higher value livestock holdings may be confounded by greater household wealth. However, our analyses were not consistent with this possibility. Associations between livestock ownership and anaemia were present even after adjusting for several socio‐demographic factors that proxy a household's wealth. Furthermore, livestock ownership typologies, TLU and total livestock quantity were not associated with asset‐based wealth. Nevertheless, there are recognized limitations to assessing socio‐economic status using an asset‐based wealth indicator (Poirier et al., 2020), and short‐term changes in income from livestock assets among livestock‐dependent households may not be captured appropriately.

Contrary to prior studies in Ghana (Christian et al., 2019; Jones et al., 2018), we did not observe positive associations between chicken ownership, or any livestock indicator, and anaemia in children. Inconsistent findings of these studies and ours may be due to differences in how livestock ownership was assessed. We assessed the distribution and quantity of livestock species in study households, whereas previous studies did not, and as noted above, the household composition of flocks and herds may be an important factor in how households can utilize their livestock. Context may also be a key factor determining how livestock can impact child anaemia. Our study, which was conducted in communities located less than 2 h from the capital city of Accra, may be different from other studies in how households depended on livestock for their livelihoods, how animals were managed and whether people had access to resources such as medical clinics, markets and veterinary services. Infectious disease outbreaks are common among free‐range poultry flocks, and recent changes to poultry ownership may also not be captured in cross‐sectional assessments.

Although our study found no evidence of a significant mediation pathway between livestock ownership, consumption of ASFs and anaemia, ownership of poultry and cattle was positively associated with consumption of chicken meat and cow's milk, respectively. Other investigations of associations between livestock ownership and child ASF consumption have found inconsistent results, including no associations (Dumas et al., 2018; Jones et al., 2018), positive associations when livestock are female owned (Jin & Iannotti, 2014) or positive associations dependent on the type and number of livestock owned (Broaddus‐Shea et al., 2020; Choudhury & Headey, 2018; de Bruyn et al., 2018; Headey & Hirvonen, 2016; Hoddinott et al., 2015; Mosites et al., 2016; Rawlins et al., 2014). Because livestock are viewed as a financial and reproductive asset, households preferentially purchase ASFs rather than source ASFs from their animals. In Ghana, as in other African countries, poultry are occasionally slaughtered for consumption, often for special occasions or guests, but larger livestock such as goats and sheep are consumed only if the animal is sick or dies (Aboe et al., 2006; Bundala et al., 2020; Colecraft et al., 2006; Haileselassie et al., 2020; Nyantakyi‐Frimpong et al., 2018). Furthermore, lack of safe processing and refrigeration in low‐income Ghanaian communities limits the potential to use livestock for meat consumption (Colecraft et al., 2006). The lack of an indirect effect of chicken meat consumption between poultry ownership and anaemia may reflect the infrequency with which children consumed chicken meat, limiting the impact on children's overall micronutrient intake. Cow's milk is also unlikely to reduce children's anaemia because unfortified cow's milk is a poor source of iron (Neumann et al., 2002).

Although there is some evidence that livestock ownership is associated with increased risk of morbidity in young children, findings are mixed and show heightened risk of diarrhoea in some countries but not others (de Bruyn et al., 2018; Headey et al., 2017; Kaur et al., 2017; Zambrano et al., 2014). Our study found no adverse associations between livestock ownership and fever, diarrhoea, cough or inflammation. In our sample, over half of households confined poultry and other livestock at night, but very few did so inside their household dwelling, which may influence the risk of exposure to the pathogens in animal faeces (Headey & Hirvonen, 2016). Livestock faeces, particularly from free‐roaming chickens, may also be widely distributed across communities, resulting in community‐level rather than household‐level exposure. In our study, livestock faeces were observed in the yards of over two‐thirds of households that reported no livestock ownership, suggesting that even children living in non‐livestock‐owning households may be exposed to pathogens from livestock faeces. Alternatively, perhaps no association between livestock and illness was found because the morbidity indicators that we used as proxies for pathogenic infection do not capture asymptomatic infections. Detection of enteropathogens in non‐diarrhoeal stool samples is common among young children in LMICs (Platts‐Mills et al., 2015), and asymptomatic enteropathogen infection has been associated with EED (Kosek & The MAL‐ED Network Investigators, 2017), linear growth faltering (Rogawski et al., 2018) and lower Hb concentrations (MAL‐ED Network Investigators, 2018). We did not find any soil‐transmitted helminth infections in children, consistent with studies in similar regions of Ghana that have found low prevalences of helminths (<5%) in children 2–5 years old (Christian et al., 2019; Forson et al., 2018). However, cattle, sheep and goats have been shown through molecular detection methods to harbour helminth species in Ghana's Greater Accra Region (Squire et al., 2018), suggesting that we may not have found helminth infections because of using a less sensitive method of detection.

Strengths of this study included detailed characterization of household livestock ownership and the use of iron and inflammatory biomarkers to examine potential pathways between livestock ownership and anaemia. However, this study had several limitations. First, it was cross‐sectional in design, which prevented us from determining a temporal link between livestock ownership and anaemia, evaluating seasonal effects or drawing any causal inferences from our findings. Second, the sample size of cattle owners (Type 5) was small, and the typologies‐based approach was underpowered to detect differences in child anaemia. Nevertheless, small sample sizes increase the likelihood of false positives; thus, power may not have been a limitation in this study because we detected a significant result even with a small number of cattle‐owning households. Still, households in Type 5 differed in several characteristics (e.g. occupation, religion and household size) from the other typologies. Despite controlling for these factors in our analyses, there may be an unmeasured factor associated with these households driving the relationship with anaemia. This limits the generalizability of our findings. We analysed several other indicators of livestock ownership, which confirmed our findings, but it is possible that these indicators, especially TLU and the total quantity of livestock, were also influenced by the limited cattle owners in the study sample. Third, we did not assess the quantity of ASFs consumed, nor was nutrient intake measured, which may explain why we did not see any mediation through diet. Lastly, although we found that livestock were kept as a financial asset, this study was not designed to explore income‐based pathways, which may be an important component in how livestock ownership affects anaemia.

Anaemia among children under 5 years old remains an intractable public health problem in many LMICs. Our findings in Ghana suggest that diversified ownership of livestock, such as cows or goats and sheep along with poultry, may be an accessible nutrition‐sensitive approach to reducing anaemia among young children, but that this may not be the result of higher ASF consumption. Rather, owning a diverse and sufficiently large herd of livestock may provide households with a financial resource that benefits their children's health. Further research elucidating non‐dietary pathways between livestock ownership and anaemia in Ghana would help to clarify how households decide to consume or sell livestock and how livestock‐derived income is used. Research on the frequency and quantity of ASFs needed to impact child micronutrient status and Hb concentrations would also help livestock development programmes identify feasibility of dietary benefits from household livestock ownership. Finally, given the negative associations found between chickens and anaemia in prior studies, further evaluation is needed to determine whether livestock‐derived infections in children increase their risk of anaemia and whether animal management strategies can mitigate such risks. Identifying the importance of financial, dietary and infectious disease pathways between livestock ownership and child anaemia will help livestock development programmes clarify policy priorities to maximize the benefits and minimize risks of livestock for low‐ and middle‐income households.

## CONFLICTS OF INTEREST

The authors declare that they have no conflicts of interest.

## CONTRIBUTIONS

NJL conceived and designed the study; MLW, AB, GF, JNSE, BA and ADJ advised on the research design; NJL and SN supervised data collection; NJL managed, cleaned and analysed the data; NJL wrote the first draft of the manuscript; all authors contributed to the writing and revision of the manuscript; all authors read and approved the final manuscript.

## Supporting information


**Supplemental Figure 1.** Map of (A) study region in Ghana and (B) study districts and communities.
**Supplemental Figure 2.** Predicted probability estimates (with 95% confidence interval) for child's consumption of (a) cow meat, (b) goat, sheep, or pig meat, (c) chicken meat, (d) organ meat, (e) chicken eggs, and (f) cow milk by household livestock typology categories, holding covariates from adjusted logistic regression models in Supplemental Table 6 constant. Animal source food consumption is modeled as dichotomous consumption of the food in the three months preceding survey administration, among children 6‐59 months old in Greater Accra Region, Ghana (n=470). Livestock typology categories: 1 – no livestock; 2 – only poultry (<12); 3 – only poultry (≥12); 4 – small livestock +/− poultry; and 5 – cattle +/− small livestock or poultry.
**Supplemental Table 1.** Distribution and number of livestock species reared by households in each livestock typology category (n=470). Values are the number (%) of households that own each livestock species and the median (range) number of livestock owned within each typology†.
**Supplemental Table 2.** Bivariate comparisons of child and household characteristics with livestock typology among children 6‐59 months old in Greater Accra Region, Ghana, October‐November 2018 (n=470). Values are % or mean ± SD.
**Supplemental Table 3.** Self‐reported sale and consumption of livestock and livestock products from own livestock holdings among livestock‐rearing households, reported by livestock category (poultry, small livestock, cattle)†.
**Supplementary Table 4.** Adjusted associations between measures of household livestock ownership and anemia, hemoglobin, and iron deficiency in children aged 6‐59 months in Greater Accra Region, Ghana, October‐November 2018 (n=470)†.
**Supplemental Table 5.** Unadjusted associations between livestock ownership typology and indicators of illness and diet among children 6‐59 months old in Greater Accra Region, Ghana, October‐November 2018 (n=470). Values are % or mean ± SD.
**Supplemental Table 6.** Multivariate logistic regression of the associations between livestock ownership typology and child consumption of animal‐source foods in the past three months in Greater Accra Region, Ghana, October‐November 2018 (n=470)†. Values are adjusted odds ratios.

## Data Availability

The data that support the findings of this study are available from the corresponding author upon reasonable request.
