# Supplementary material for: Associations between livestock ownership and lower odds of anaemia among children 6–59 months old are not mediated by animal‐source food consumption in Ghana
Source: Matern Child Nutr. 2021 Mar 1;17(3):e13163. doi: 10.1111/mcn.13163 (PMC8189243; doi:10.1111/mcn.13163)
Supplement: Supplementary file 1 — Supplemental Figure 1. Map of (A) study region in Ghana and (B) study districts and communities. Supplemental Figure 2. Predicted probability estimates (with 95% confidence interval) for child's consumption of (a) cow meat, (b) goat, sheep, or pig meat, (c) chicken meat, (d) organ meat, (e) chicken eggs, and (f) cow milk by household livestock typology categories, holding covariates from adjusted logistic regression models in Supplemental Table 6 constant. Animal source food consumption is modeled as dichotomous consumption of the food in the three months preceding survey administration, among children 6‐59 months old in Greater Accra Region, Ghana (n=470). Livestock typology categories: 1 – no livestock; 2 – only poultry (<12); 3 – only poultry (≥12); 4 – small livestock +/− poultry; and 5 – cattle +/− small livestock or poultry. Supplemental Table 1. Distribution and number of livestock species reared by households in each livestock typology category (n=470). Values are the number (%) of households that own each livestock species and the median (range) number of livestock owned within each typology†. Supplemental Table 2. Bivariate comparisons of child and household characteristics with livestock typology among children 6‐59 months old in Greater Accra Region, Ghana, October‐November 2018 (n=470). Values are % or mean ± SD. Supplemental Table 3. Self‐reported sale and consumption of livestock and livestock products from own livestock holdings among livestock‐rearing households, reported by livestock category (poultry, small livestock, cattle)†. Supplementary Table 4. Adjusted associations between measures of household livestock ownership and anemia, hemoglobin, and iron deficiency in children aged 6‐59 months in Greater Accra Region, Ghana, October‐November 2018 (n=470)†. Supplemental Table 5. Unadjusted associations between livestock ownership typology and indicators of illness and diet among children 6‐59 months old in Greater Accra Region, Ghana, October‐Novemb [file MCN-17-e13163-s001.docx]

**Associations between livestock ownership and lower odds of anemia among children 6-59 months old are not mediated by animal-source food consumption in Ghana.**

Lambrecht et al.

**
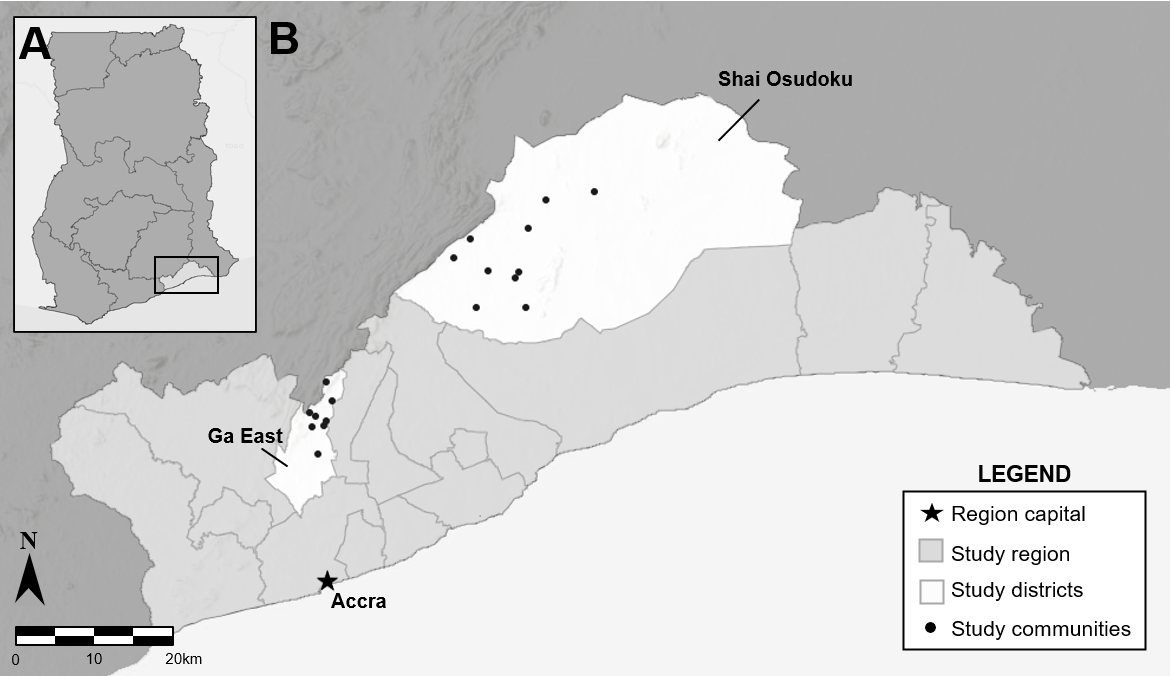
**

**Supplemental Figure 1.** Map of (A) study region in Ghana and (B) study districts and communities.

**
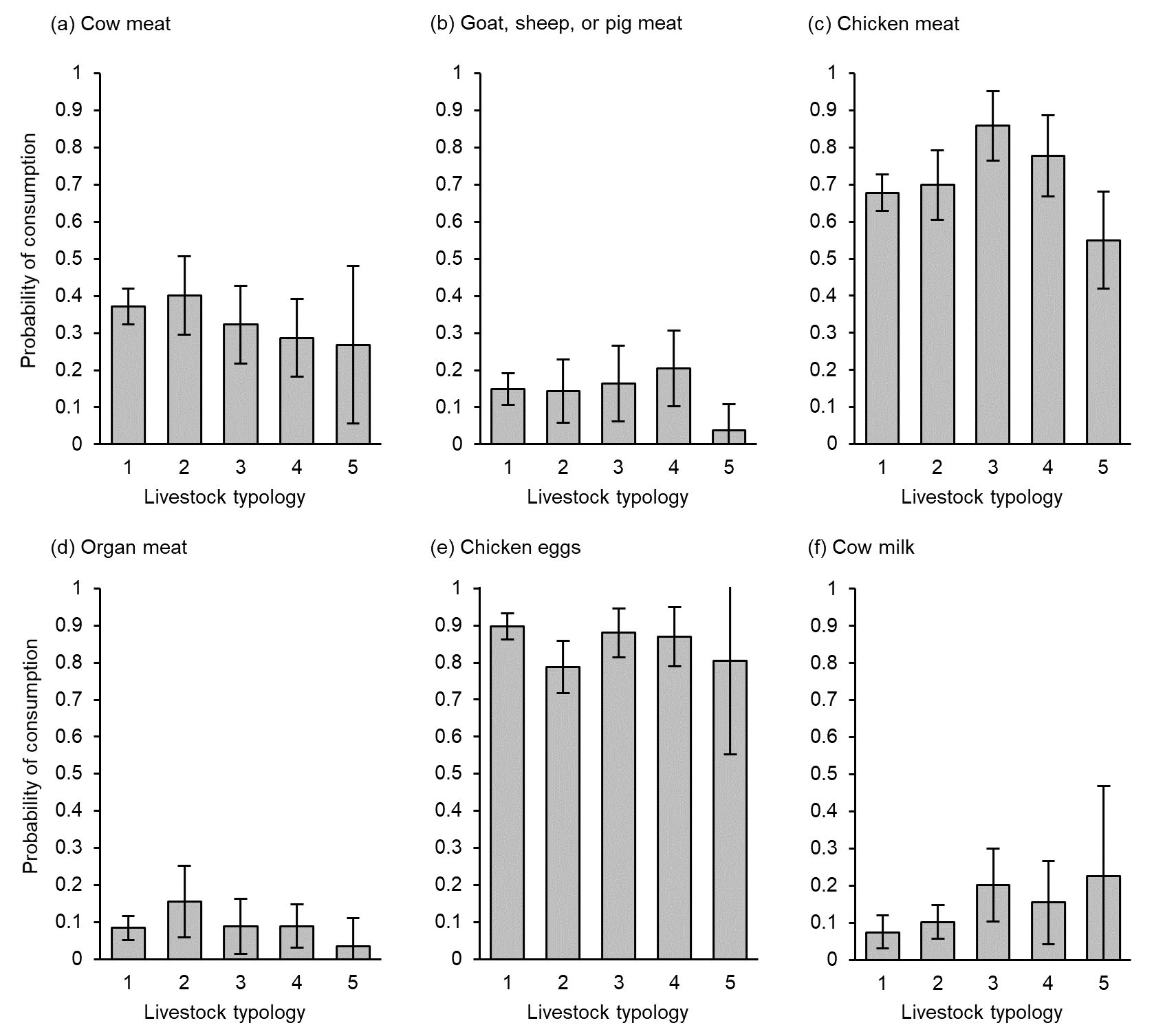
**

**Supplemental Figure 2.** Predicted probability estimates (with 95% confidence interval) for child’s consumption of (a) cow meat, (b) goat, sheep, or pig meat, (c) chicken meat, (d) organ meat, (e) chicken eggs, and (f) cow milk by household livestock typology categories, holding covariates from adjusted logistic regression models in Supplemental Table 6 constant. Animal source food consumption is modeled as dichotomous consumption of the food in the three months preceding survey administration, among children 6-59 months old in Greater Accra Region, Ghana (n=470). Livestock typology categories: 1 – no livestock; 2 – only poultry (<12); 3 – only poultry (≥12); 4 – small livestock +/- poultry; and 5 – cattle +/- small livestock or poultry.

**Supplemental Table 1.** Distribution and number of livestock species reared by households in each livestock typology category (n=470). Values are the number (%) of households that own each livestock species and the median (range) number of livestock owned within each typology^†^.

|  | **Type 1** | **Type 2** | | **Type 3** | | **Type 4** | | **Type 5** | |
| --- | --- | --- | --- | --- | --- | --- | --- | --- | --- |
|  | **No livestock** | **Poultry (<12)** | | **Poultry (≥12)** | | **Small livestock +/- poultry** | | **Cattle +/- small livestock or poultry** | |
|  | **(n=265)** | **(n=71)** | | **(n=59)** | | **(n=60)** | | **(n=15)** | |
|  | Number (%) | Number (%) | Median (range) | Number (%) | Median (range) | Number (%) | Median (range) | Number (%) | Median (range) |
| Any livestock | 0 (0%) | 71 (100%) | 5 (1-11) | 59 (100%) | 20 (12-50) | 60 (100%) | 20 (2-175) | 15 (100%) | 90 (20-212) |
| Chickens | ∙ | 71 (100%) | 5 (1-11) | 58 (98.3%) | 20 (0-50) | 49 (81.7%) | 11 (0-50) | 14 (93.3%) | 12 (0-60) |
| Ducks | ∙ | 0 (0%) | 0 | 5 (8.5%) | 0 (0-32) | 8 (13.3%) | 0 (0-20) | 3 (20.0%) | 0 (0-27) |
| Guinea fowl | ∙ | 1 (1.4%) | 0 (0-4) | 2 (3.4%) | 0 (0-21) | 4 (6.7%) | 0 (0-40) | 4 (26.7%) | 0 (0-15) |
| Turkeys | ∙ | 0 (0%) | 0 | 0 (0%) | 0 | 2 (3.3%) | 0 (0-10) | 1 (6.7%) | 0 (0-5) |
| Goats | ∙ | 0 (0%) | 0 | 0 (0%) | 0 | 43 (71.7%) | 2 (0-60) | 6 (40.0%) | 0 (0-50) |
| Sheep | ∙ | 0 (0%) | 0 | 0 (0%) | 0 | 13 (21.7%) | 0 (0-40) | 6 (40.0%) | 0 (0-45) |
| Pigs | ∙ | 0 (0%) | 0 | 0 (0%) | 0 | 6 (10.0%) | 0 (0-41) | 2 (13.3%) | 0 (0-10) |
| Grasscutter | ∙ | 0 (0%) | 0 | 0 (0%) | 0 | 4 (6.7%) | 0 (0-12) | 0 (0%) | 0 |
| Rabbits or rats | ∙ | 0 (0%) | 0 | 0 (0%) | 0 | 2 (3.3%) | 0 (0-4) | 0 (0%) | 0 |
| Cattle | ∙ | 0 (0%) | 0 | 0 (0%) | 0 | 0 (0%) | 0 | 15 (100%) | 50 (8-120) |
| TLU, median (min-max)^‡^ | 0 | 0.05 (0.01-0.13) | | 0.24 (0.12-0.98) | | 0.73 (0.10-10.65) | | 38.60 (5.72-85.02) | |
| ^†^Multiple species of livestock may be present in a single household; thus, the categories of species are not mutually exclusive.  ^‡^TLU was calculated as the sum of the number of each livestock species multiplied by its weight conversion factor (0.70 for cattle, 0.20 for pigs, 0.10 for goats and sheep, 0.03 for ducks, turkeys, and guinea fowl, 0.02 for grasscutter, rabbits, and rats, and 0.01 for chickens).  Abbreviations: TLU – tropical livestock unit score | | | | | | | | | |

**Supplemental Table 2.** Bivariate comparisons of child and household characteristics with livestock typology among children 6-59 months old in Greater Accra Region, Ghana, October-November 2018 (n=470). Values are % or mean ± SD.

| **Indicator** | **Type 1** | **Type 2** | **Type 3** | **Type 4** | **Type 5** | **P-value^†^** |  |
| --- | --- | --- | --- | --- | --- | --- | --- |
|  | **No livestock** | **Poultry (<12)** | **Poultry (≥12)** | **Small livestock +/- poultry** | **Cattle +/- small livestock or poultry** |  |  |
| Child characteristics |  |  |  |  |  |  |  |
| Female sex, % | 57.4 | 43.7 | 45.8 | 46.7 | 46.7 | 0.15 |  |
| Age (6-23 months old), % | 55.5 | 45.1 | 54.2 | 38.3 | 33.3 | 0.06 |  |
| Malaria parasitemia, % | 9.8 | 7.0 | 5.1 | 6.7 | 13.3 | 0.67 |  |
| Household characteristics |  |  |  |  |  |  |  |
| Household size | 4.5 ± 1.5 | 4.9 ± 2.0 | 5.7 ± 2.3 | 5.8 ± 2.0 | 7.3 ± 2.9 | **<0.001** |  |
| Number of children <5y | 1.3 ± 0.5 | 1.4 ± 0.6 | 1.3 ± 0.5 | 1.4 ± 0.7 | 1.5 ± 0.8 | 0.94 |  |
| Head of household sex (female), % | 24.2 | 28.2 | 11.9 | 20.0 | 0.0 | **0.04** |  |
| Head of household religion, % |  |  |  |  |  | **<0.001** |  |
| Christian | 88.3 | 85.9 | 88.1 | 81.7 | 46.7 |  |  |
| Muslim | 9.1 | 11.3 | 10.2 | 13.3 | 53.3 |  |  |
| Other | 2.6 | 2.8 | 1.7 | 5.0 | 0.0 |  |  |
| Head of household ethnic group, % |  |  |  |  |  | **<0.001** |  |
| Ga-Dangme | 43.8 | 38.0 | 33.9 | 55.0 | 33.3 |  |  |
| Akan | 20 | 16.9 | 11.9 | 3.3 | 0.0 |  |  |
| Ewe | 24.2 | 33.8 | 39 | 28.3 | 13.3 |  |  |
| Other | 12.1 | 11.3 | 15.3 | 13.3 | 53.3 |  |  |
| Head of household occupation, % |  |  |  |  |  | **<0.001** |  |
| Unemployed/unpaid work | 9.4 | 11.3 | 11.9 | 8.3 | 0.0 |  |  |
| Agricultural labor | 10.9 | 16.9 | 25.4 | 33.3 | 93.3 |  |  |
| Self-employed | 64.9 | 63.4 | 55.9 | 51.7 | 6.7 |  |  |
| Salaried employment | 14.7 | 8.5 | 6.8 | 6.7 | 0.0 |  |  |
| Maternal education, % |  |  |  |  |  | **<0.001** |  |
| None or nursery | 18.1 | 11.3 | 30.5 | 28.3 | 60 |  |  |
| Primary | 23.0 | 32.4 | 18.6 | 21.7 | 0.0 |  |  |
| Junior | 42.3 | 32.4 | 45.8 | 45.0 | 33.3 |  |  |
| Senior or higher | 16.6 | 23.9 | 5.1 | 5.0 | 6.7 |  |  |
| Access to electricity, % | 87.2 | 84.5 | 88.1 | 91.7 | 86.7 | 0.81 |  |
| Access to improved water source, % | 97.7 | 95.8 | 100 | 93.3 | 93.3 | 0.19 |  |
| Type of sanitation, % |  |  |  |  |  | **0.006** |  |
| Flush or pour flush | 14.3 | 2.8 | 13.6 | 16.7 | 6.7 |  |  |
| Pit latrine | 62.3 | 62.0 | 50.9 | 48.3 | 33.3 |  |  |
| Open defecation | 23.4 | 35.2 | 35.6 | 35.0 | 60.0 |  |  |
| Mud floor, % | 1.5 | 0.0 | 0.0 | 0.0 | 13.3 | **0.001** |  |
| Thatch roof, % | 0.4 | 2.8 | 0.0 | 1.7 | 6.7 | 0.07 |  |
| Asset-based wealth quintile, % |  |  |  |  |  | 0.90 |  |
| Lowest | 18.5 | 28.2 | 13.6 | 15.0 | 26.7 |  |  |
| Low | 18.9 | 22.5 | 23.7 | 23.3 | 20.0 |  |  |
| Middle | 21.5 | 16.9 | 20.3 | 25 | 13.3 |  |  |
| High | 20.8 | 18.3 | 23.7 | 18.3 | 20.0 |  |  |
| Highest | 20.4 | 14.1 | 18.6 | 18.3 | 20.0 |  |  |
| Asset-based wealth quintile | 3.1 ± 1.4 | 2.7 ± 1.4 | 3.1 ± 1.3 | 3.0 ± 1.3 | 2.9 ± 1.6 | 0.32 |  |
| District, % |  |  |  |  |  | **<0.001** |  |
| Shai Osudoku | 50.9 | 67.6 | 69.5 | 60.0 | 100.0 |  |  |
| Ga East | 49.1 | 32.4 | 30.5 | 40.0 | 0.0 |  |  |
| ^†^P- values calculated from chi-square statistics for comparisons of proportions and F-statistics using ANOVA for comparisons of means. | | | | | | | |

**Supplemental Table 3.** Self-reported sale and consumption of livestock and livestock products from own livestock holdings among livestock-rearing households, reported by livestock category (poultry, small livestock, cattle)^†^.

|  | **Households with poultry** | | **Households with small livestock** | | **Households with cattle** | | |
| --- | --- | --- | --- | --- | --- | --- | --- |
|  | **(n=193)** | | **(n=67)** | | **(n=15)** | | |
|  | **Sold^‡^** | **Consumed** | **Sold^‡^** | **Consumed** | **Sold^‡^** | **Consumed** | |
| *Percent of households that sold or consumed livestock/livestock products from own livestock holdings* | | | | | | | |
| Poultry (chickens, ducks, turkeys, guinea fowl), in past 3 months | 18.1% | 38.3% | ∙ | ∙ | ∙ | | ∙ |
| Poultry eggs^§^, in past 3 months | 1.0% | 37.8% | ∙ | ∙ | ∙ | | ∙ |
| Small livestock (goats, sheep, pigs), in past 12 months | ∙ | ∙ | 34.3% | 19.4% | ∙ | | ∙ |
| Cattle, in past 12 months | ∙ | ∙ | ∙ | ∙ | 40.0% | | 6.7% |
| Cow’s milk^¶^, in milk-producing months | ∙ | ∙ | ∙ | ∙ | 86.6% | | 60.0% |
| *Mean (range) number of livestock that were sold or consumed per household* | | | | | | | |
| Poultry (chickens, ducks, turkeys, guinea fowl), in past 3 months | 6 (1-35) | 3 (1-10) | ∙ | ∙ | ∙ | | ∙ |
| Small livestock (goats, sheep, pigs), in past 12 months | ∙ | ∙ | 5 (1-30) | 2 (1-6) | ∙ | | ∙ |
| Cattle, in past 12 months | ∙ | ∙ | ∙ | ∙ | 4 (1-7) | | 1 (1) |

^†^Households may own multiple categories of animals (e.g., 33% of households with poultry also own other livestock). Households that own more than one category of livestock are represented within each category of livestock that they own.

^‡^Average self-reported income from sales of live poultry in this sample was GHC 40 ($7) per animal. Live poultry sales were made up of 77% chickens, 10% ducks, 3% turkeys, and 10% guinea fowl. Average self-reported income from sales of live small livestock (goats, sheep, pigs) in this sample was GHC 342 ($61) per animal. Live small livestock sales were made up of 52% goats, 28% sheep, and 20% pigs. Average self-reported income from sales of live cattle in this sample was GHC 1,572 ($281) per animal.

^§^Poultry birds laid on average 15 eggs per week. Households that consumed eggs (n=73) reported consuming on average 7 eggs per week in the past 3 months. Eggs for consumption were primarily sourced from chickens (92%), rather than ducks (3%), turkeys (1%), or guinea fowl (4%).

^¶^Of households with cattle that milked their cows in the past 12 months (n=14), one household reported consuming “all or nearly all (90-100%)” of the milk produced, three households reported consuming “less than half (10-40%)” of the milk produced, five reported consuming “a small amount (1-10%)” of the milk produced, and five reported that they “did not consume” any of the milk produced.

**Supplementary Table 4.** Adjusted associations between measures of household livestock ownership and anemia, hemoglobin, and iron deficiency in children aged 6-59 months in Greater Accra Region, Ghana, October-November 2018 (n=470)^†^.

| Indicator | **Anemia** | | **Hemoglobin (g/dL)** | | **Low SF** | | **High sTfR** | |
| --- | --- | --- | --- | --- | --- | --- | --- | --- |
|  | OR | 95% CI | β | 95% CI | OR | 95% CI | OR | 95% CI |
| Ownership of any livestock (yes/no) | 0.88 | (0.67, 1.15) | 0.03 | (-0.20, 0.26) | 0.97 | (0.64, 1.49) | 1.03 | (0.69, 1.54) |
| Total number of livestock owned among livestock owners (log-2 scale)^‡^ | **0.80*** | (0.67, 0.95) | 0.14^+^ | (-0.00, 0.27) | 0.98 | (0.89, 1.10) | 0.99 | (0.89, 1.10) |
| TLU | **0.97*** | (0.95, 1.00) | 0.01 | (-0.00, 0.02) | 0.98 | (0.95, 1.02) | 1.00 | (0.98, 1.02) |
| TLU (log-2 scale)^‡^ | **0.77**** | (0.66, 0.91) | 0.09 | (-0.04, 0.23) | 0.87 | (0.65, 1.17) | 0.98 | (0.80, 1.20) |
| Ownership of cattle (yes/no)^§^ | 0.42^+^ | (0.17, 1.02) | 0.32 | (-0.44, 1.09) | 0.38 | (0.08, 1.78) | 0.95 | (0.42, 2.13) |
| Number of cattle owned among cattle owners (log-2 scale)^‡^ | **0.86*** | (0.74, 0.99) | 0.05 | (-0.07, 0.17) | 0.83 | (0.63, 1.11) | 1.03 | (0.89, 1.20) |
| Ownership of goats, sheep, or pigs (yes/no)^§^ | 0.58^+^ | (0.32, 1.06) | **0.39*** | (0.10, 0.69) | 0.85 | (0.44, 1.64) | 0.92 | (0.52, 1.61) |
| Number of goats, sheep, or pigs owned among goat, sheep, and pig owners (log-2 scale)^‡^ | **0.86*** | (0.74, 1.00) | 0.08 | (-0.02, 0.18) | 1.00 | (0.82, 1.24) | 0.94 | (0.77, 1.16) |
| Ownership of poultry (yes/no)^§^ | 1.09 | (0.79, 1.51) | -0.10 | (-0.36, 0.15) | 1.08 | (0.69, 1.67) | 1.10 | (0.69, 1.75) |
| Number of poultry owned among poultry owners (log-2 scale)^‡^ | 0.91 | (0.76, 1.09) | 0.09 | (-0.04, 0.23) | 0.95 | (0.85, 1.07) | 0.93 | (0.74, 1.16) |
| Livestock typology |  |  |  |  |  |  |  |  |
| Type 1 – No livestock (reference) | - | - | - | - | - | - | - | - |
| Type 2 – Poultry (<12) | 1.14 | (0.74, 1.76) | -0.14 | (-0.79, 0.21) | 0.97 | (0.56, 1.69) | 1.22 | (0.68, 2.18) |
| Type 3 – Poultry (≥12) | 1.07 | (0.63, 1.82) | -0.07 | (-0.44, 0.31) | 1.27 | (0.81, 2.01) | 0.92 | (0.44, 1.90) |
| Type 4 – Small livestock +/- poultry | 0.62^+^ | (0.36, 1.07) | 0.24 | (-0.06, 0.55) | 0.87 | (0.43, 1.78) | 0.95 | (0.55, 1.66) |
| Type 5 – Cattle +/- small livestock or poultry | **0.32**** | (0.14, 0.71) | 0.48 | (-0.29, 1.24) | 0.36 | (0.08, 1.64) | 0.95 | (0.38, 2.38) |
| Livestock typology (ordinal) | **0.86*** | (0.75, 0.99) | 0.07 | (-0.03, 0.16) | 0.94 | (0.78, 1.14) | 0.98 | (0.82, 1.18) |
| ^†^Models adjusted for child age and sex, malaria, number of children under 5 in household, sex of head of household, head of household ethnic group and religion, maternal education, type of toilet facility, asset-based wealth quintile, and district. Robust standard errors are adjusted for community cluster. Marginal significance (0.05≤p<0.1) indicated by ^+^. Statistical significance indicated by * for p<0.05, ** for p<0.01, and *** for p<0.001. ^‡^Log-2 scaled effect estimates are interpreted as the change in the outcome variable for a two-fold increase in the number of livestock owned.  ^§^Controlling for ownership of other livestock species (i.e., cattle; goats, sheep, or pigs; poultry).  Abbreviations: SF – serum ferritin; sTfR – serum transferrin receptor; TLU – tropical livestock unit score | | | | | | | | |

**Supplemental Table 5.** Unadjusted associations between livestock ownership typology and indicators of illness and diet among children 6-59 months old in Greater Accra Region, Ghana, October-November 2018 (n=470). Values are % or mean ± SD.

|  | **Type 1** | **Type 2** | **Type 3** | **Type 4** | **Type 5** | **P-value^¶^** |
| --- | --- | --- | --- | --- | --- | --- |
|  | **No livestock** | **Poultry (<12)** | **Poultry (≥12)** | **Small livestock +/- poultry** | **Cattle +/- small livestock or poultry** |  |
|  | **(n=265)** | **(n=71)** | **(n=59)** | **(n=60)** | **(n=15)** |  |
| *Illness indicators* |  |  |  |  |  |  |
| Fever in past 7 days, % | 23.8 | 35.2 | 18.6 | 15.0 | 20.0 | 0.07 |
| Diarrhea in past 7 days, % | 9.8 | 7.0 | 8.5 | 0.0 | 0.0 | 0.09 |
| Cough/cold in past 7 days, % | 24.2 | 26.8 | 18.6 | 15.0 | 20.0 | 0.46 |
| Inflammation (CRP>5mg/L), % | 17.7 | 11.3 | 15.2 | 15.0 | 13.3 | 0.75 |
| Inflammation (AGP>1g/L), % | 34.7 | 31.0 | 40.7 | 35.0 | 53.3 | 0.48 |
| *Dietary Indicators* |  |  |  |  |  |  |
| Dietary diversity score^†^ | 3.4 ± 1.6 | 3.4 ± 1.4 | 3.7 ± 1.4 | 3.5 ± 1.7 | 3.5 ± 2.0 | 0.67 |
| ASF consumption in past 24 hours |  |  |  |  |  |  |
| Cow meat, % | 4.9 | 2.8 | 5.1 | 6.7 | 6.7 | 0.88 |
| Other red meat, % | 0.8 | 1.4 | 0.0 | 3.3 | 0.0 | 0.40 |
| Chicken meat, % | 16.2 | 19.7 | 15.3 | 15.0 | 0.0 | 0.45 |
| Organ meats, % | 0.4 | 1.4 | 1.7 | 0.0 | 0.0 | 0.65 |
| Eggs, % | 29.1 | 16.9 | 28.8 | 23.3 | 33.3 | 0.28 |
| Animal milk^‡^, % | 20.0 | 14.1 | 22.0 | 23.3 | 40.0 | 0.22 |
| ASF consumption in past 3 months |  |  |  |  |  |  |
| Cow meat, % | 36.6 | 38.0 | 30.5 | 31.7 | 40.0 | 0.83 |
| Cow meat frequency, times/month | 1.6 ± 3.2 | 1.6 ± 3.2 | 1.6 ± 3.4 | 1.2 ± 3.3 | 2.3 ± 4.9 | 0.83 |
| Goat meat, % | 10.2 | 12.7 | 8.5 | 11.7 | 6.7 | 0.92 |
| Goat meat frequency, times/month | 0.3 ± 1.2 | 0.4 ± 1.5 | 0.3 ± 1.3 | 0.4 ± 1.6 | 0.1 ± 0.3 | 0.88 |
| Sheep meat, % | 1.5 | 4.2 | 0.0 | 5.0 | 0.0 | 0.20 |
| Sheep meat frequency, times/month | 0.0 ± 0.4 | 0.0 ± 0.3 | 0.0 ± 0.0 | 0.1 ± 1.0 | 0.0 ± 0.0 | 0.54 |
| Pig meat, % | 4.2 | 0.0 | 3.4 | 8.3 | 0.0 | 0.15 |
| Pig meat frequency, times/month | 0.1 ± 0.5 | 0.0 ± 0.0 | 0.1 ± 0.5 | 0.3 ± 1.2 | 0.0 | 0.09 |
| Chicken meat, % | 66.0 | 73.2 | 84.8 | 80.0 | 60.0 | **0.017** |
| Chicken meat frequency, times/month | 5.1 ± 6.2 | 4.9 ± 5.2 | 5.0 ± 4.5 | 4.2 ± 4.0 | 2.7 ± 3.6 | 0.46 |
| Chicken eggs, % | 89.1 | 81.7 | 88.1 | 88.3 | 80.0 | 0.46 |
| Chicken eggs frequency, times/month | 8.4 ± 6.7 | 7.3 ± 7.1 | 8.0 ± 7.5 | 6.9 ± 4.7 | 7.1 ± 8.3 | 0.48 |
| Cow milk^§^, % | 6.4 | 11.3 | 18.6 | 18.3 | 46.7 | **<0.001** |
| Cow milk frequency, times/month | 0.6 ± 3.1 | 0.1 ± 0.5 | 1.0 ± 2.8 | 1.9 ± 8.4 | 13.1 ± 19.2 | **<0.001** |
| ^†^Dietary diversity score ranges from 0 to 7 food groups consumed in the past 24 hours.  ^‡^Animal milk includes fresh milk, tinned milk, or powdered milk.  ^§^Cow milk includes only fresh cow’s milk.  ^¶^P- values calculated from chi-square statistics for comparisons of proportions and F-statistics using ANOVA for comparisons of means.  Abbreviations: ASF – animal-source food; CRP – C-reactive protein; AGP – α-1-acid glycoprotein | | | | | | |

**Supplemental Table 6.** Multivariate logistic regression of the associations between livestock ownership typology and child consumption of animal-source foods in the past three months in Greater Accra Region, Ghana, October-November 2018 (n=470)^†^. Values are adjusted odds ratios.

| Indicator | Cow meat | Goat, sheep, or pig meat | Chicken meat^‡^ | Organ meats | Chicken eggs | Cow milk |
| --- | --- | --- | --- | --- | --- | --- |
| Livestock ownership typology (Ref: No livestock) | - | - | - | - | - | - |
| Type 2 – Poultry (<12) | 1.15 | 0.95 | 1.13 | 2.21 | **0.36*** | 1.54 |
| Type 3 – Poultry (≥12) | 0.77 | 1.14 | **3.47**** | 1.06 | 0.81 | **4.39**** |
| Type 4 – Small livestock +/- poultry | 0.64 | 1.58 | 1.84 | 1.07 | 0.72 | **2.86*** |
| Type 5 – Cattle +/- small livestock or poultry | 0.57 | 0.18 | 0.50^+^ | 0.36 | 0.41 | **5.32*** |
| Child sex (Ref: Male) |  |  |  |  |  |  |
| Female | **1.58*** | 1.57^+^ | **1.66*** | **1.70*** | 0.71 | 0.83 |
| Child age (Ref: 24-59 months) |  |  |  |  |  |  |
| 6-23 months | **0.28***** | **0.31***** | **0.14***** | 0.49^+^ | **0.13***** | **0.22***** |
| Number of children under 5y in household | 1.08 | 1.47 | 1.18 | 0.98 | 0.87 | 0.89 |
| Head of household sex (Ref: Male) |  |  |  |  |  |  |
| Female | 1.19 | 1.18 | 1.73^+^ | 0.58 | 2.25 | 1.74 |
| Head of household religion (Ref: Christian) |  |  |  |  |  |  |
| Muslim | 1.79 | 2.66 | 0.91 | 1.28 | 0.50 | **8.43**** |
| Traditional or no religion | 0.27 | **5.11*** | 3.53^+^ | 1.51 | 0.36^+^ | 0.72 |
| Head of household ethnic group (Ref: Ga-Dangme) |  |  |  |  |  |  |
| Akan | 0.75 | 1.02 | 1.06 | 2.81^+^ | 2.34^+^ | 0.14^+^ |
| Ewe | 0.70^+^ | 0.68 | 1.49 | 1.65 | **3.27**** | 0.88 |
| Other | 2.00^+^ | 2.32 | 1.89^+^ | 3.10 | 4.29 | 0.49 |
| Maternal education (Ref: None or nursery) |  |  |  |  |  |  |
| Primary | 1.00 | 1.48 | 1.39 | **0.33*** | 1.52 | 1.46 |
| Junior | 1.41 | 1.00 | 1.07 | 0.39^+^ | 0.94 | 1.09 |
| Senior or higher | 1.58 | **4.45*** | 1.70 | **0.14*** | 1.47 | 0.58 |
| Asset-based wealth quintile (Ref: Lowest) |  |  |  |  |  |  |
| Low | **2.04*** | 0.77 | 1.62 | 2.67 | **2.62*** | 0.54 |
| Middle | **2.46**** | 1.61 | 1.21 | **6.94**** | **3.64**** | 0.80 |
| High | **2.44*** | 1.28 | 1.28 | **6.06***** | **3.65**** | 1.80 |
| Highest | **3.85***** | 2.61 | 1.61 | **15.82***** | 2.13 | 0.67 |

^†^Animal-source food consumption variables are dichotomous (yes/no) for any consumption in the past three months. Models adjusted for listed covariates. Robust standard errors are adjusted for community cluster. Marginal significance (0.05≤p<0.1) indicated by ^+^. Statistical significance indicated by * for p<0.05, ** for p<0.01, and *** for p<0.001.

^‡^The category for chicken meat includes consumption of other poultry meats (turkey, guinea fowl, and duck meat) in the past three months. 99.7% of children consumed chicken meat, 2.1% of children consumed other poultry meat in addition to chicken meat, and 0.3% of children consumed other poultry meat but not chicken meat in the past three months.
